# Measuring changes in perception using the Student Perceptions of Physician-Pharmacist Interprofessional Clinical Education (SPICE) instrument

**DOI:** 10.1186/1472-6920-14-101

**Published:** 2014-05-20

**Authors:** Joseph A Zorek, Eric J MacLaughlin, David S Fike, Anitra A MacLaughlin, Mohammed Samiuddin, Rodney B Young

**Affiliations:** 1 Pharmacy Practice Division, University of Wisconsin-Madison School of Pharmacy, 1016 Rennebohm Hall, 777 Highland Avenue, Madison, WI 53705-2222, USA; 2 Department of Pharmacy Practice, Texas Tech University Health Sciences Center (TTUHSC) School of Pharmacy, Amarillo, TX, USA; 3 Department of Family Medicine, TTUHSC School of Medicine, Amarillo, TX, USA; 4 Department of Internal Medicine, TTUHSC School of Medicine, Amarillo, TX, USA; 5 School of Graduate Studies and Research, University of the Incarnate Word, San Antonio, TX, USA; 6 Hereford Pharmacy LLC, Hereford, TX, USA

**Keywords:** Interprofessional education, Interprofessional collaborative practice, Interprofessional teamwork, Interprofessional relations, Professional roles, Pretest-posttest

## Abstract

**Background:**

The Student Perceptions of Physician-Pharmacist Interprofessional Clinical Education (SPICE) instrument contains 10 items, 3 factors (interprofessional teamwork and team-based practice, roles/responsibilities for collaborative practice, and patient outcomes from collaborative practice), and utilizes a five-point response scale (1 = strongly disagree, 5 = strongly agree). Given the SPICE instrument’s demonstrated validity and reliability, the objective of this study was to evaluate whether it was capable of measuring changes in medical (MS) and pharmacy students’ (PS) perceptions following an interprofessional education (IPE) experience.

**Methods:**

In this prospective cohort study, MS and PS completed the SPICE instrument before and after participation in a predefined IPE experience. Descriptive statistics were used to characterize students and pre-post responses. Independent samples t tests and Fisher’s Exact tests were used to assess group difference in demographic variables. Mann Whitney U tests were used to assess between-group differences in item scores. Wilcoxon Signed-Rank tests were used to evaluate post-participation changes in item scores. Spearman correlations were calculated to assess associations between ordinal demographic variables and item scores, and whether the number of clinic visits completed was associated with post-test responses. Paired samples t tests were used to calculate mean score changes for each of the factors.

**Results:**

Thirty-four MS and 15 PS were enroled. Baseline differences included age (25.3. ± 1.3 MS *vs.* 28.7 ± 4.4 PS; p = 0.013), years full-time employment (0.71 ± 0.97 MS *vs.* 4.60 ± 4.55 PS; p < 0.001), and number of prior IPE rotations (1.41 ± 1.74 MS *vs.* 3.13 ± 2.1 PS; p < 0.001). Two items generated baseline differences; 1 persisted post-participation: whether MS/PS should be involved in teamwork (3.91 MS *vs.* 4.60 PS; p < 0.001). For all students, significant mean score increases were observed for role clarity (“my role” [3.72 *vs.* 4.11; p = 0.001] and “others’ roles” [3.87 *vs.* 4.17; p = 0.001]), impact of teamwork on patient satisfaction (3.72 *vs.* 4.34; p < 0.001), and ideal curricular location for IPE (4.06 *vs.* 4.34; p = 0.002). Significant increases were observed for all three factors (teamwork, p = 0.003; roles/responsibilities and patient outcomes, p < 0.001).

**Conclusions:**

This study demonstrated the SPICE instrument’s ability to measure changes in perception for medical and pharmacy students exposed to an IPE experience, both at the individual item level and at the factor level.

## Background

An international consensus has emerged in support of interprofessional education (IPE) as a mechanism to ensure health professionals are prepared to improve health outcomes through team-based delivery of care. The World Health Organization proposed the following definition for IPE in 2010, which has subsequently been adopted worldwide: *“when students from two or more professions learn about, from, and with each other to enable effective collaboration and improve health outcomes”*[1]. Building on this work, the Canadian Interprofessional Health Collaborative and the Interprofessional Education Collaborative in the United States developed competency frameworks to guide the design and evaluation of IPE experiences [2,3]. While independently developed, both frameworks have an explicit patient-centered focus and propose similar domains of interest, including domains dedicated to interprofessional communication, roles/responsibilities, teamwork, and values/ethics. These frameworks have become authoritative sources for educators designing curricular experiences aimed at preparing health professional students for interprofessional collaborative practice (IPCP).

Accrediting bodies within the health professions have also begun incorporating IPE-specific standards into their accreditation documents [4-6]. This has further motivated academic administrators to create and assess IPE experiences in order to demonstrate compliance with accreditation standards. As a result, the importance of valid and reliable IPE measurement instruments has become evident. Importantly, the National Center for Interprofessional Practice and Education in the United States released a compilation of IPE measurement instruments that may be used for this purpose [7]. This compilation currently includes 28 instruments spanning 6 categories (attitudes; behavior; knowledge, skills, abilities; organizational practice; other; patient satisfaction; and provider satisfaction). These instruments vary widely. For example, the number of items within the instruments range from 10 to 59, the response scales range from 4- to 10-points, and the number of factors (i.e., subscales) range from 1 to 12. The diversity of factors represented within these instruments is of particular interest. Educators must select a measurement instrument that matches their student population (i.e., one that was validated in their population of interest), contains factors relevant to the outcomes they desire, and fits logistically into their educational environment (e.g., didactic *vs.* experiential, etc.).

A recent webinar hosted by the National Center describing results of a study investigating assessment and evaluation in IPE acknowledged the lack of a theoretical basis for most published instruments, and simultaneously stressed the need for longitudinal studies documenting students’ progress via repeated measurements [8]. To satisfy accreditation mandates for high quality IPE, administration of a measurement instrument at two or more points in time has the potential to generate data demonstrating progression/growth attributable to programmatic or curricular design. With the aforementioned Canadian and United States-based competency frameworks essentially filling the theoretical void within the field, it is therefore important for educators to utilize measurement instruments designed to assess domains highlighted within these frameworks.

The authors created the Student Perceptions of Physician-Pharmacist Interprofessional Clinical Education (SPICE) instrument guided by the Interprofessional Education Collaborative’s competency framework. The SPICE instrument contains 10 items and 3 factors dedicated to interprofessional teamwork and team-based practice, roles/responsibilities for collaborative practice, and patient outcomes from collaborative practice. Responses are captured via a five-point Likert-type scale (1 = strongly disagree, 5 = strongly agree). The SPICE instrument was designed for experiential education settings involving IPE experiences between medical and pharmacy students, with the explicit desire to produce a convenient, quick tool for experiential educators practicing and teaching in busy health care settings. A detailed description of the development and validation of the SPICE instrument has been previously described [9].

Having demonstrated the validity and reliability of the SPICE instrument, and in consideration of a growing desire within the academy to conduct longitudinal assessments of students via repeated measurements, the primary objective of this study was to assess whether the SPICE instrument was capable of measuring changes in medical and pharmacy students’ perceptions following an IPE experience. To explore this capacity, the SPICE instrument was administered to a small sample of medical students (MS) and pharmacy students (PS) at Texas Tech University Health Sciences Center (TTUHSC), a public institution located in Texas, USA, before and after participation in an IPCP clinic.

## Methods

### Study design

The TTUHSC Institutional Review Board approved this prospective cohort study. Third year MS and fourth year PS were recruited to participate in an IPCP clinic led by physician and pharmacist faculty members. Recruitment was limited to third year MS and fourth year PS due to profession-specific experiential education schedules. The clinic was housed within the TTUHSC Center for Family Medicine and was designed specifically to deliver preventive care services reimbursable through Medicare, a government-run health program for United States citizens over the age of 65 [9,10]. Immediately following enrolment and informed consent, but prior to exposure to the clinic, students completed two paper-based data collection forms: (1) a demographic questionnaire (Table 1), and (2) a pre-test consisting of the 10-item SPICE instrument (Table 2). Student participation in the study was terminated upon completion of a post-test consisting of the same 10-item SPICE instrument plus an additional question (item 11), which asked students how many clinic visits they had completed.

**Table 1:** Student demographics

**Demographic variable**	**Medical (N = 34)**	**Pharmacy (N = 15)**	**p-value**^★^
	**N (%)**	**N (%)**	
Sex			1.000
Male	17 (50)	8 (53.3)	
Female	17 (50)	7 (46.7)	
Age (years) [mean ± SD]	25.3 ± 1.3	28.7 ± 4.4	***0.013***
Race			0.538^†^
White	14 (41.2)	8 (53.3)	
Black	2 (5.9)	0 (0)	
Hispanic	2 (5.9)	3 (20)	
Asian	15 (44.1)	3 (20)	
Other	1 (2.9)	1 (6.7)	
Year in school			**<** ***0.001***
Third	32 (94.1)	0 (0)	
Fourth	2 (5.9)	15 (100)	
Percent current year completed			0.538^‡^
0-25	7 (20.6)	6 (40)	
26-50	7 (20.6)	2 (13.3)	
51-75	13 (38.2)	2 (13.3)	
76-100	7 (20.6)	5 (33.3)	
Highest degree earned			***<0.001***^***§***^
None	0 (0)	6 (40)	
Associates	0 (0)	1 (6.7)	
Baccalaureate	29 (85.3)	7 (46.7)	
Masters	5 (14.7)	1 (6.7)	
Years full-time employment [mean ± SD]	0.71 ± 0.97	4.60 ± 4.55	***<0.001***
Prior rotations involving interprofessional teamwork (number) [mean ± SD]	1.41 ± 1.74	3.13 ± 2.1	***<0.001***

^★^Results demonstrating statistical significance (i.e., p ≤ 0.05) appear in bolded and italicized font.

^†^Due to small cell counts, Fisher’s Exact was applied to “White” *versus* “Non-white” to calculate this p-value.

^‡^Due to small cell counts, Fisher’s Exact was applied to “0-50%” *versus* “51-100%” to calculate this p-value.

^§^Due to small cell counts, Fisher’s Exact was applied to “Baccalaureate or higher” *versus* “Less than baccalaureate” to calculate this p-value.

**Table 2:** **Comparison of pre- to post-test average scores**^★^

**No.**	**Survey item**	**Between-group**			**Between-group**			**All students**^**†**^		
		**Pre-test averages**			**Post-test averages**			**Pre/post-test averages**		
		**MS**^**†**^	**PS**^**†**^	**p-value**^**‡**^	**MS**^**†**^	**PS**^**†**^	**p-value**^**‡**^	**Pre**	**Post**	**p-value**^**‡**^
01	Working with another discipline of students enhances my education	4.15	4.40	0.176	4.41	4.60	0.257	4.26	4.47	0.008
02	My role within the interdisciplinary team is clearly defined	3.71	3.67	0.903	4.09	4.13	0.887	3.72	4.11	***0.001***
03	Health outcomes are improved when patients are treated by a team of professionals from different disciplines	4.26	4.60	0.079	4.47	4.67	0.264	4.40	4.53	0.109
04	Patient satisfaction is improved when patients are treated by a team of professionals from different disciplines	3.68	3.80	0.648	4.19	4.67	0.023	3.72	4.34	***<0.001***
05	Participating in educational experiences with another discipline of students enhances my future ability to work on an interdisciplinary team	4.26	4.47	0.312	4.28	4.67	0.047	4.34	4.40	0.532
06	All health professional students should be educated to establish collaborative relationships with members from other disciplines	4.29	4.53	0.157	4.34	4.73	0.047	4.38	4.47	0.285
07	I understand the roles of other professionals within the interdisciplinary team	3.71	4.20	0.017	4.09	4.33	0.159	3.87	4.17	***0.001***
08	Clinical rotations are the ideal place within their respective curricula for medical and pharmacy students to interact	3.88	4.40	0.006	4.16	4.73	***0.003***	4.06	4.34	***0.002***
09	Physicians and pharmacists should collaborate in teams	4.00	4.60	***0.002***	4.25	4.73	0.007	4.21	4.40	0.013
10	During their education, medical and pharmacy students should be involved in teamwork in order to understand their respective roles	3.82	4.47	***<0.001***	3.91	4.60	***<0.001***	4.04	4.13	0.346

^★^Based on 5-point, Likert-type responses whereby 5 = Strongly agree, 4 = Agree, 3 = Neutral, 2 = Disagree, and 1 = Strongly disagree. ^†^All students, N = 49; medical students (MS), N = 34; pharmacy students (PS), N = 15. ^‡^A Bonferroni correction for multiple tests was performed which set *alpha* for significance at ≤0.005. Results demonstrating statistical significance appear in bolded and italicized font.

### Interprofessional education experience

After a brief orientation by the pharmacist faculty member, MS and PS were paired and instructed to work together to review electronic medical records of scheduled patients with a focus on preventive care services needed and potential or actual medical/medication-related problems. The pharmacist and students then interviewed scheduled patients, documenting preventive care histories and updated electronic medical records. Medical students led assessments of fall history, depression, activities of daily living, and independent activities of daily living. Pharmacy students led a comprehensive medication history and review. The pharmacist and students then discussed their findings and recommendations with an attending physician. Together as a team, the physician, pharmacist, and students developed a care plan that they communicated to the patient collectively. Patients were provided a written list of preventive care- and medication-related recommendations.

### Data analysis

Descriptive statistics were used to characterize self-reported demographic variables of students and their responses on the pre- and post-test SPICE instrument. To test for group differences (e.g., MS *vs.* PS) in demographic variables, independent samples t tests and Fisher’s Exact tests were used. To assess between-group differences in instrument item scores, Mann Whitney U tests were used. Wilcoxon Signed-Rank tests were conducted to determine pre-to-post change in item scores. Spearman correlations were calculated to test the association of ordinal demographic variables with students’ responses (i.e., item scores). Spearman correlations were used to assess whether the number of completed clinic visits was associated with post-test responses. Finally, changes in perception for each of the three factors was calculated using a paired-samples t test. The level of significance was *alpha* = 0.05. A Bonferroni correction for multiple tests was performed to control for Type I error inflation. For this study’s sample size (N = 49), alpha = 0.05, and a standardized effect size (d = 0.41), a two-tailed, paired-samples t test will achieve power of 80%.

## Results

Thirty-four MS and 15 PS completed the study. As Table 1 demonstrates, the groups did not differ in terms of sex, race, or percent current year completed. They did differ, however, on the other five demographic variables collected. These included age, year in school, highest degree earned, years full-time employment, and number of prior rotations involving interprofessional teamwork. On average, the PS were roughly three years older than the MS and had nearly four more years of full-time work experience.

The differences observed between groups on year in school and prior IPCP rotations can be attributed to the scheduling issues described above; specifically, fourth year MS and third year PS were excluded from participation in the IPCP clinic shortly after enrolment opened. It was expected that PS would have more IPE experience at baseline given their additional year of education. The difference in highest degree earned was also expected due to differences in admissions requirements. The decision was made to evaluate potential between-group differences in response to the IPE experience due to these expected demographic differences.

Some notable pre- and post-test between-group differences were observed. At baseline, significant differences were observed between groups for two items relating to whether (1) physicians and pharmacists should collaborate in teams (Table 2, Item 9), and (2) MS and PS should work in teams during their education (Item 10). PS responses were significantly higher for these items.

Of the two items that demonstrated significant differences at baseline, only 1 persisted after the IPE experience: whether MS and PS should work in teams (Item 10). While scores for this item increased for both groups following the IPE experience, the magnitude of difference remained similar. The margin of difference between MS and PS for Item 9 (whether physicians and pharmacists should collaborate in teams) decreased following the IPE experience such that statistical significance was lost.

In evaluating changes in scores following the IPE experience for all students (N = 49), a mean score increase was observed for all of the items in the instrument (Table 2). Of these, statistically significant increases were observed for the following four items: (1) understanding my role (Item 2), (2) impact of IPCP on patient satisfaction (Item 4), (3) understanding others’ roles (Item 7), and (4) clinical rotations as ideal place within curricula for IPE (Item 8).

At baseline, MS agreed or strongly agreed (i.e., mean score ≥4) with 50% (5/10) of items within the instrument. Following the IPE experience, this percentage increased to 90% (9/10). Using the same metric, a 20% increase was observed for PS (80% [8/10] pre-test vs. 100% [10/10] post-test). When considering mean score ≥4 for all students, 70% (7/10) met this criteria at baseline, while 100% (10/10) did so following the IPE experience.

Results from Mann Whitney U tests demonstrated no statistically significant differences in response to any of the 10 items based on academic discipline, year in school, or sex. With the exception of number of previous IPCP rotations, results of Spearman correlations to evaluate associations between change in scores and the remainder of the demographic variables were non-significant. For the single significant result, the number of previous IPCP rotations was negatively correlated with change score for understanding roles within the team (r_S_ = -0.46, p = 0.001). In other words, as the number of IPCP rotations increased, the magnitude of change from pre- to post-test for this item decreased.

Factor scores were calculated as the mean of item scores within the factor. Changes in factor scores from pre- to post-IPE experience were evaluated. Significant changes were observed for each of the three factors (Table 3). Student perceptions were significantly more positive following the IPE experience. Standardized effect sizes for the three factors ranged from 0.46-0.71 (Table 3), indicating moderate to large effects.

**Table 3:** **Comparison of pre- to post-test factor scores**^★^

**Factor**	**Pre**	**Post**	**Change**	**p-value**	**d**^**†**^
Teamwork and team-based practice	4.24 ± 0.41	4.40 ± 0.46	0.16 ± 0.34	0.003	0.46
Roles/responsibilities for collaborative practice	3.79 ± 0.54	4.14 ± 0.49	0.35 ± 0.52	<0.001	0.68
Patient outcomes from collaborative practice	4.07 ± 0.57	4.45 ± 0.51	0.38 ± 0.54	<0.001	0.71

^★^Paired-samples t test, Mean ± SD.

^†^Cohen’s d standardized effect size.

## Discussion

A series of Cochrane Collaboration review articles evaluating the effects of IPE on professional practice and health care outcomes published since 2001 highlight the evolving evidence base for IPE [11-13]. The most recent review identified 15 studies of sufficient methodological rigor to meet inclusion criteria [13]. This represented a marked increase from previous iterations (2001, 0 studies; 2008, 6 studies). Positive outcomes were demonstrated in seven studies, mixed outcomes (positive and neutral) in four studies, and no impact in the remaining studies. The authors’ main conclusion has remained stable throughout the series; specifically, the high variability of IPE interventions and outcomes measured in the included studies preclude their ability to draw generalizable inferences about the effectiveness of IPE.

While the evidence base for IPE continues to grow, the benefits associated with team-based health care delivery are becoming well documented [14-23]. Specific studies that inspired the creation of the IPCP clinic at TTUHSC demonstrated gains in blood pressure control by physician-pharmacist teams [16,20], improvement in quality of care [22], and increases in patient satisfaction alongside decreases in health care costs [23]. Demonstrating the value of team-based health care delivery is critical, and may even supersede the need to demonstrate the value of IPE initiatives via rigorous research methodologies. The assertion that curricula responsible for educating health professional students should incorporate opportunities to learn within a team-based approach is supported if teams of health professionals working collaboratively can improve health outcomes and the experience patients have interfacing with the health care system while decreasing health care costs.

Interprofessional competency development is necessary to guide health professional educators as they attempt to develop and incorporate IPE curricular elements, which underscores the importance of the aforementioned Canadian- and United States-based frameworks [2,3]. Increasing health professional students’ understanding of the various roles and responsibilities for different members of the interprofessional team is a core competency of both frameworks. It was thus encouraging to observe in this study statistically significant increases in mean scores for the roles/responsibilities for collaborative practice factor within the SPICE instrument. This finding supports the notion that the IPCP clinic at TTUHSC is a valuable practice site for MS and PS to learn about, from, and with one another. It also provides evidence that the SPICE instrument can be used in a pre-/post-test manner within a longitudinal study to evaluate progress related to this important competency.

The finding that the number of prior IPCP rotations was negatively correlated with change score for understanding roles within the team also supports the notion that IPE initiatives can impact students’ perceptions related to IPCP. This finding confirmed that students with more IPE experience would be further along in their IPE development than their less experienced peers. It also lends credence to calls within the academy for longitudinal studies involving repeated measurements to evaluate progress.

Statistically significant increases in mean scores for the teamwork and team-based practice factor, and the patient outcomes from collaborative practice factor, provide further evidence of the SPICE instrument’s ability to capture change. Given the emphasis within the academy on interprofessional teamwork and the implicit understanding that team-based care improves patient outcomes, this finding provides evidence that the SPICE instrument is capable of detecting and tracking students’ perceptions related to both.

Like the SPICE instrument, the Attitudes Toward Health Care Teams (ATHCT) scale is an IPE measurement instrument with a strong focus on teamwork [24]. A revised version of this instrument (ATHCT-R), published by Hyer *et al*., is most comparable to the SPICE instrument, as it has been used to assess health professional students’/trainees’ attitudes toward team learning and teamwork [25,26]. The ATHCT-R instrument is composed of 21 items using a 6-point Likert-type response scale. It contains three factors intended to measure attitudes toward (1) team value, (2) team efficiency, and (3) physician’s shared role. Leipzig *et al*. utilized the ATHCT-R instrument to assess attitudes of trainees in medicine, advanced practice nursing, and social work towards IPCP [26]. The authors found an overall positive disposition toward teamwork within this population, while also noting several between-group differences (e.g., physician trainees were less positively inclined towards IPCP than the nurse practitioner and social work trainees). This general pattern was observed in the present study, as well.

Researchers have also administered the ATHCT-R instrument before and after exposure to an IPE experience as an assessment mechanism akin to the methodology employed in the present study. Fulmer *et al*. utilized this tool to measure the impact of the Geriatric Interdisciplinary Team Training program on 537 health professional students representing 20 different professions [27]. The authors of this study observed statistically significant improvements in attitudes across the three ATHCT-R factors irrespective of profession. Similar to Leipzig *et al*., they also reported differences between professions. More recently, Curran *et al*. evaluated the impact of a workshop developed to improve interprofessional collaborator skills in a sample of 82 participants, which included pre-licensure medical residents and a variety of post-licensure allied health professionals (e.g., nurses, social workers, occupational therapists, etc.) [28]. The investigators administered a 14-item version of the ATHCT instrument in a pre-/post-test study design and reported a significant improvement in pre- to post- overall mean score change for the pre-licensure medical residents.

The one item that demonstrated statistically significant between-group differences before and after the IPE experience described in the present study warrants further discussion in relation to the studies just described. This item asked whether MS and PS should be involved in teamwork during their education (Item 10). Mean scores for PS were significantly higher than for MS. The margin of difference remained stable from pre- to post-test, with PS rating this item much higher than MS. This finding is similar to those described from studies utilizing the various versions of the ATHCT instrument. It is possible that this reflects certain practice realities. For example, pharmacists are reliant on collaborations with physicians in order to impact patient care to the fullest extent. It is also possible that physicians may view IPE negatively within the context of pharmacists’ desires to expand their scope of practice [29,30]. Effective interprofessional communication and a synergistic IPE/IPCP design, such as the TTUHSC preventive care clinic described herein, may mitigate this issue. Given the potential for improved health outcomes, it is certainly worth the time and effort.

There are several limitations to this study that warrant discussion. First, the hours of operation of the IPCP clinic dictated the quantity and type of MS and PS eligible for participation. The vast majority of MS enroled were in their third year of school, while all PS enroled were in their fourth year. Curricular restrictions for each of the professional programs led to the majority of MS spending a single day in the clinic while the majority of PS were exposed to the clinic on multiple occasions. As a result, there were over twice as many MS enroled in the study, as well as differences in the number of clinic visits completed between the groups. It was encouraging, therefore, that significant positive change in response scores were observed following the IPE experience despite these curricular restrictions. Additionally, given the small sample size, multivariable analyses controlling for covariates were not conducted. Large-scale studies should be conducted to confirm the findings from this study. The unique nature of the IPE experience and the single site setting also limit the generalizability of the findings. Despite these limitations, this study provided interesting and informative assessment feedback on the effects of an IPCP clinic on students’ perceptions using repeated measurement with the SPICE instrument.

## Conclusions

The Student Perceptions of Physician-Pharmacist Interprofessional Clinical Education (SPICE) instrument consists of 10 items and 3 factors dedicated to interprofessional teamwork and team-based practice, roles/responsibilities for collaborative practice, and patient outcomes from collaborative practice. This study demonstrated the SPICE instrument’s ability to measure changes in perception for medical and pharmacy students exposed to an IPE experience, both at the individual item level and at the factor level. The SPICE instrument may be used by educators, administrators, and researchers in longitudinal studies involving repeated measurements of student perceptions. Further research involving the SPICE instrument is warranted to (1) assess its external validity in a broad population of medical and pharmacy students, (2) evaluate test-retest reliability and criterion validity, and (3) expand this instrument for use in other health professions.

## Abbreviations

IPCP: Interprofessional collaborative practice; IPE: Interprofessional education; MS: Medical students; PS: Pharmacy students; SPICE: Student Perceptions of Physician-Pharmacist Interprofessional Clinical Education; TTUHSC: Texas Tech University Health Sciences Center.

## Competing interests

The authors declare that they have no competing interests.

## Authors’ contributions

JZ, EM, AM, and DF conceived and designed this study while JZ was a pharmacotherapy resident and assistant clinical instructor at Texas Tech University Health Sciences Center. JZ recruited patients for participation in the clinic. EM coordinated pharmacy and medical students’ recruitment. EM and AM obtained informed consent from patients and students. EM, AM, MS, and RY provided clinical services and served as preceptors for students. JZ managed the data collection and entry processes. JZ and DF performed data analysis. JZ wrote the first draft of the manuscript. All authors contributed to the critical revision of the first draft and approved the final manuscript for publication.

## Pre-publication history

The pre-publication history for this paper can be accessed here:

http://www.biomedcentral.com/1472-6920/14/101/prepub
